# The Best Supportive Care in Stage III Non-Small-Cell Lung Cancer

**DOI:** 10.3390/curroncol31010012

**Published:** 2023-12-29

**Authors:** Thiago Bueno de Oliveira, Debora Maloni Nasti Fontes, Tatiane Caldas Montella, Jairo Lewgoy, Carolina Dutra, Thais Manfrinato Miola

**Affiliations:** 1 Medical Oncology Department, AC Camargo Cancer Center, São Paulo 01509-001, Brazil; 2 Nurse Navigator Department, AC Camargo Cancer Center, São Paulo 01509-001, Brazil; debora.nasti@accamargo.org.br; 3 Medical Oncology Department, Oncoclínicas, Rio de Janeiro 22250-905, Brazil; tatiane.montella@medicos.oncoclinicas.com; 4 Medical Oncology Department, Hospital Mãe de Deus, Porto Alegre 90880-481, Brazil; jairo.lewgoy@maededeus.com.br; 5 Medical Oncology Department, Clínica Soma, Florianópolis 88020-210, Brazil; carolina.dutra@ynovapesquisa.com; 6 Nutritional Oncology Department, AC Camargo Cancer Center, São Paulo 01509-001, Brazil; thais.miola@accamargo.org.br

**Keywords:** multidisciplinary teams, non-small-cell lung cancer, mapping review, chemoradiotherapy, adverse events, immune checkpoint inhibitors

## Abstract

Lung cancer is a major cause of cancer deaths worldwide. Non-small-cell lung cancer (NSCLC) represents most lung cancer cases, and approximately one-third of patients present with stage III disease at diagnosis. As multiple treatment plans can be adopted for these patients depending on tumor size and nodal staging, stage III NSCLC management is challenging. Over the past decades, multidisciplinary teams (MDTs) have been implemented in healthcare services to coordinate actions among the different health care professionals involved in cancer care. The aim of this review was to discuss real-world evidence of the impact of MDTs on stage III NSCLC management, survival, and quality of life. Here, we performed a literature review to investigate the role of nutrition and navigational nursing in NSCLC care and the influence of MDTs in the choice of treatment plans, including immunotherapy consolidation, and in the management of chemotherapy and radiotherapy-related adverse events. We also performed a mapping review to identify gaps in the implementation of cancer care MDTs in healthcare services around the world.

## 1. Introduction

Lung cancer accounts for 11.4% (2.206 million) of total cancer cases and 18% (1.796 million) of total cancer deaths worldwide, making it the most frequent cause of cancer deaths among men and the third among women [1]. Non-small-cell lung cancer (NSCLC) is the most frequent form of lung cancer, responsible for 85% of cases in the US. Optimal staging is crucial for disease management once treatment recommendations are stage-specific for NSCLC [2].

Approximately one-third of patients with NSCLC present with Stage III disease [3]. This stage of NSCLC is the most heterogeneous and challenging to manage. There are two main routes of therapy: surgical-based versus nonoperative; for the former, neoadjuvant chemotherapy or chemoradiotherapy are part of the treatment for most cases, followed by resection. This article is focused on unresectable NSCLC and will not discuss neoadjuvant approaches. The standard of care for unresectable NSCLC is concomitant chemoradiation (cCRT) followed by consolidation immunotherapy with durvalumab [2,4].

Over the past several decades, MDT-based approaches have been implemented in healthcare services around the world with the objective of improving triage, diagnosis, supportive care, treatment adherence, and adverse event management [5]. Lung cancer MDTs usually are formed by medical oncologists, radiation oncologists, radiologists, thoracic surgeons, nurses, nutritionists, psychologists, and other health professionals that may fill the needs of the team [6].

## 2. Methodology

The present article aims to review and discuss the evidence in the literature about the impact of MDT-based approaches on the improvement of outcomes and management of unresectable stage III NSCLC. The authors joined together to carry out a narrative literature review that aimed to collect existing evidence on the best supportive care in stage III NSCLC management in clinical practice with MDTs. Based on clinical experience on the management of cases of stage III NSCLC within a MDT model, the authors met online in July 2021 to identify and discuss relevant topics. This article addresses the following selected issues: (i) the importance of MDTs in stage III NSCLC management; (ii) cCRT in the context of MDTs; (iii) monitoring of adverse events; (iv) immunotherapy consolidation.

As MDTs are gaining momentum in NSCLC clinical practice, the authors also decided to perform a comprehensive mapping review search about the selected topics on Cochrane Library, EMBASE, Lilacs, PubMed, and Web of Science up to May 18th, 2023. The PICO (population, intervention, comparator, and outcomes) question and mapping review search eligibility criteria are described in Supplementary Materials (Methodology S1. Mapping review; Figure S1. PRISMA flow chart of search strategy divided by identification, screening, eligibility, and inclusion; Methodology S2. Search strategy). The assistance of an independent scientific consultancy agency (Corebox, Brazil) was sought in order to guarantee the scientific accuracy of the mapping review and the medical writing process. The entire project was supported by an unrestricted and unconditional grant from AstraZeneca.

## 3. Results

### 3.1. Multidisciplinary Team (MDT) Approach in the Management of Stage III Non-Small-Cell Lung Cancer

#### 3.1.1. MDTs in Cancer Care

The complexity of cancer management has increased with molecular and genomic biology developments over the past 20 years [7]. These advances have significant consequences for health services. Currently, cancer care requires coordination between a wide range of health care professions such as oncologists, surgeons, radio-oncologists, nutritionists, and nurses. These MDTs are an alternative approach to the standard care model and fundamentally support a timely and accurate diagnosis [8].

MDTs are an essential component of cancer care for various tumors. A systematic review showed a survival benefit among breast, head and neck, colorectal, esophageal, and lung cancer [9].

#### 3.1.2. MDTs and NSCLC—Impact of MDTs on Survival

Evidence supporting the usefulness of MDTs in lung cancer is growing, especially regarding specific types and stages of the disease [6,10,11,12,13,14,15]. MDTs can also significantly reduce treatment costs [16]. One early investigation of patients with inoperable NSCLC in the UK compared survival between 117 patients treated in a newly implemented MDTs and 126 that received a standard model of care. The study found improved survival for MDTs [17]. The first studies to assess the effect of MDTs in NSCLC that provide reasonable evidence that MDT care is significantly associated with better survival rates are presented in Table 1. MDTs had a better 1-year survival rate compared to those who were not discussed (18% vs. 8%, respectively; *p* < 0.006) (OR 0.48; 95% CI: 0.25–0.92).

### 3.2. The Role of MDTs on the Treatment of Unresectable Stage III NSCLC

#### 3.2.1. Nutritional Support and Physical Activity

Malnutrition is highly prevalent in cancer care, as 70% of these patients are considered to be malnourished [43]. In a real-world cohort of stage III NSCLC, more than 20% of cases presented ≥10% weight loss at initial evaluation [44]. During cCRT treatment, a worsening of weight loss is expected, putting the patient at greater risk for treatment complications and worse outcomes [45].

The European Society for Clinical Nutrition and Metabolism guidelines strongly recommends nutritional screening to detect nutritional problems early in patient management. In the abnormal screening patients, a quantitative assessment of nutritional parameters such as nutritional intake, body composition, and physical performance is planned [46]. Sarcopenia when present implies special nutritional care, once associated with a three-fold increased death risk in lung cancer. The overall prevalence of sarcopenia in a series of NSCLC was 46.8% with computed tomography (CT). It is interesting to note that only 7.5% of patients were underweight (BMI < 18.5) [47]. Furthermore, a relation between sarcopenia and poor clinical outcomes has been demonstrated in surgical and cCRT treatments [29]. Energy requirements vary in cancer according to the primary site, disease stage, and physical activity level. For practical purposes, experts assume that the total energy expenditure is similar to that of healthy people (25–30 kcal/kg/day) [46].

Dietary counseling (DC) supports cancer treatment to help patients stay healthy and avoid infections and disease recurrence. A nutritional and metabolic approach inside an MDT is emerging as a best practice to help maintain the patient’s nutrition and delay the onset of cancer cachexia. Such an approach may improve the tolerability of aggressive treatments like cCRT [48]. Most experts recommend DC, even though this intervention is rarely seen in lung cancer studies, its benefit is reported in other tumors [49].

Exercise is the best intervention to minimize muscle loss and should be included in the patient treatment plan [50]. The benefits of physical activity are well-known in cancer patients [51]. The decline of physical activities is prevalent during and after cancer treatment (e.g., major surgery, cCRT), which can aggravate sarcopenia. Seven days of bed rest causes a muscle mass loss of 1 kg loss in elderly, and it takes 12 weeks of endurance exercise to regain 1.5 kg of lean muscle mass in these individuals [50]. This decline is potentialized in lung cancer once other important comorbidities (e.g., chronic obstructive pulmonary disease) are present in more than 50% of patients in the diagnostic [52].

Nutritional interventions and physical activity improve cancer outcomes [53]. The group presents the evidence on dietary counseling, oral nutritional supplements (ONS), enteral, and parenteral nutrition, as well as the use of omega three and protein supplementation for cancer patients, and the clinical data of the benefits of physical activity interventions in Supplementary Table S1.

#### 3.2.2. Navigation Nursing

Navigation is the act of guiding a patient through the healthcare system. A patient navigator, usually a nurse, acts as a bridge between the patient and the healthcare system. They promote timely access to screening programs, diagnostic services, facilitate doctor visits, and help avoid delays and redundancies by developing effective and straightforward clinical pathways. Most importantly, nurse navigators avoid patient mistrust of the medical system and loss of follow-up by providing clear communication about ongoing procedures in close contact with patients, alleviating anxiety and fear [5]. Successful navigation systems have already been implemented to improve care with HIV, diabetes, and cardiovascular disease [30].

Cancer care greatly benefits from navigational nurses inside a multidisciplinary context. Therefore, after navigation nurses were implemented, doctors had valuable diagnostic information at the time of the first consultation. Another single-center retrospective cohort with 308 lung cancer patients evaluated if a cancer care coordination program conducted by an advanced practice nurse had an impact on the timeliness of care.

In a multivariate linear regression analysis, the program resulted in a 23-day reduction in the interval from the first abnormal image to diagnosis (*p* < 0.016) and a 25-day reduction in the interval from the first abnormal image to treatment initiation (*p* < 0.015) [54].

Nurse navigators also enhance cancer care experience, supportive care, and satisfaction [55]. A qualitative survey with 3,278 cancer patients reported that the group attended by navigation nurses gave a higher frequency of positive ratings on all cancer care and nursing function categories [56]. Indeed, a retrospective chart study demonstrated that oncology navigational nurse visits were able to lower inpatients’ level of distress scores (*p* < 0.1046), although the sample size was small [57].

As discussed, patient navigation is a valuable tool in providing efficient cancer care. The navigation approach provides clinical pathways that reduce the interval between the entrance of the patient to the healthcare facility, diagnosis, and the start of treatment. Early availability of molecular results helps MDTs determine the optimal treatment for these patients and better manage cases. By providing patients with constant communication translated into an appropriate language, navigators alleviate psychosocial distress, clarify doubts, collect additional information, and help patients stay compliant to the treatment. The navigator integrates collected information among members of the MDT, leading to the observed improvements in the timeliness of care that further reduces patient anxiety and distress.

#### 3.2.3. Nursing Care

Besides navigation nurses guiding patients through the healthcare system, nurse practitioners are in constant contact with cancer patients. Inside a medical team, nurse practitioners are the professionals in closest proximity to the patients. The nursing consultation has three steps to be followed: physical examination, care specifics, and the orientation of NSCLC patients [32]. Indeed, patients usually develop a relationship of confidence with their nurses, as they accompany and guide patients during chemotherapy and radiotherapy sessions. Hence, oncology nurse practitioners are able to identify early changes in patient mood or behavior and play a key role in the symptom management of patients undergoing therapy. By constantly monitoring patients, they can detect signs and symptoms of potential adverse events that a patient may be experiencing during therapy as a consequence of the toxicity of chemoradiotherapy protocols for stage III NSCLC. This constant monitoring allows the medical team to adopt early interventions [58]. Details about adverse event management will be discussed in the following sections. In lung cancer patients, nurse practitioners can observe the respiratory pattern and evaluate the need for oxygen therapy, identify the presence of cyanosis, skin pallor and dyspnea [32].

Nurse practitioners also help to promote quality of life changes by preventing treatment regimen delays and avoiding more serious complications and hospitalizations. Results of a retrospective chart review with 151 stage III or IV oropharyngeal cancer patients that compared a group who received weekly nurse visits for physical examination to a group that received written and verbal education prior to starting therapy and one visit in the middle of radiation therapy observed a reduction in hospitalization rates (12% vs. 28%) and greater completion rates of the full chemotherapy regimen (90% vs. 46%) in the group that received weekly nurse visits [59]. These results highlight the potential role of good nursing care practice in promoting better quality of life for cancer patients and providing valuable clinical information for MDTs, which contributes to improving patient management and care.

### 3.3. Impact of MDTs on cCRT

Concurrent chemoradiotherapy (cCRT) is the standard of care for patients with unresectable stage III NSCLC and a good performance status. The results of several studies documenting beneficial interactions between radiation and chemotherapy demonstrated the superiority of cCRT over sequential chemoradiotherapy and it is superior in terms of survival to both sequential CRT (sCRT) and single-modality radiotherapy [60]. The choice of chemotherapy can additionally be important to the outcome of cCRT as well as the plan of radiotherapy treatment. The recommended cCRT regimens are summarized in Table S3.

An MDT approach can enable accurate assessment of patients with unresectable stage III NSCLC from the time of diagnosis, and determine the optimal treatment strategy to minimize risks and toxicity, and mainly, improve response. It is important to consider that stage III NSCLC demands challenging and complex multimodal treatment management that requires integrative knowledge on the prognostic and predictive factors of the patient population. Tumors in this stage can be resectable or not, and further choices must be made regarding whether a patient with an unresectable tumor can tolerate the potential toxicity of cCRT or needs to receive an alternative, potentially less toxic regimen, such as sequential CRT [14].

The literature reports that MDTs promote the reduction of time between the first consult, the start of therapy, impact the patient management, and increase the patient’s chance of receiving cCRT. A retrospective study with 3799 stage III NSCLC patients analyzed prediction factors associated with the receipt of cCRT. Seeing three types of specialists (medical oncologist, radiation oncologist, and surgeon) prior to the initiation of treatment increased the odds of receiving cCRT compared to seeing only one type of specialist (logistic regression OR 1.87; *p* < 0.001) [61]. A single-center study evaluated the effectiveness of an MDT approach on the outcomes of 109 patients. The time from the first physician consultation to the start of treatment was lower for patients who were referred to an MDT compared to non-MDT (mean 19.85 days versus 29.09 days, respectively; *p* = 0.043), even though the sample size was small and the result was close to the limit of significance [62]. Furthermore, in a cohort that analyzed 308 NSCLC patients, among those scheduled to receive cCRT, 89.4% started therapy within seven days of one another in the MDT group compared to 62.6% in the non-MDT group (*p* < 0.01) [63]. A national quality survey conducted by the Association of Community Cancer Centers (ACCC) identified that the frequency of MDT meetings negatively correlated with time to complete disease staging (*p* = 0.03) [64]. These results indicate that MDTs may impact the reduction of time until receipt of treatment, although more robust studies are necessary to address this matter.

A real-world study evaluated the responses of a survey from European thoracic expert opinions (*n* = 11). For stage III patients, concurrent CRT (cCRT) is the most common form of treatment, accounting for roughly 70% of cases. Performance status is used as a universal criterion for cCRT eligibility, and ICI consolidation is started within six weeks after RT completion. Durvamulab expenditures are reimbursed in all countries but with some limiting criteria, such as PD-L1 ≥ 1% and cCRT. Experts agree that therapies at durvamulab progression depend on progression timing. The authors conclude that standardized evidence-based decisions and healthcare provision are needed due to high heterogeneity in real-world practices [65].

MDTs can also provide optimized cCRT delivery and better supportive care that allows more patients to be fit for cCRT, even if they have more comorbidities [66]. In this regard, a single-center study analyzed outcomes of 89 patients with stage III NSCLC, of which 37 had some degree of comorbidity. The MDT discussed all cases before the commencement of treatment. For patients who were considered fit to undergo cCRT, the presence or absence of comorbidities was associated with similar OS (mean 18.2 versus 20.3 months, respectively; *p* = 0.803) [67]. Indeed, a retrospective study with 10,072 patients demonstrated that stage III NSCLC patients treated with cCRT at high volume facilities (HVF) experienced decreased risk of death when compared to low volume facilities (HR = 0.91; 95% CI: 0.84–0.99; *p* = 0.04), despite significantly more patients with comorbidities being admitted to HVFs [68]. Considering that cCRT provides better survival rates than sCRT, patients with comorbidities are directly benefited by the presence of MDTs. They are more likely to receive cCRT and adequate support during the course of treatment when their case is evaluated by MDTs, which may significantly increase the OS for these patients.

Due to the heterogeneity of the disease, MDTs are currently considered to be essential to determine both staging and optimal treatment of stage III NSCLC [11]. Therefore, the findings above highlight the role of MDTs in providing the expertise to promote timely, coordinated management and diagnosis that could potentially contribute to improving the quality of life and treatment indications for patients with stage III NSCLC.

#### 3.3.1. Chemotherapy Component

Platinum-based chemotherapy is the standard of care for patients with stage III NSCLC undergoing cCRT. However, the optimal regimen has not been clearly established. The two most commonly used regimens in the US are the combination of cisplatin plus etoposide (CE) and carboplatin plus paclitaxel (CP) (Supplementary Table S2) [69,70].

To date, clinical trials have not demonstrated the clear superiority of one regimen over the other. A phase III trial with 191 patients comparing CE versus CP with concurrent thoracic radiotherapy in unresectable stage III NSCLC observed that CE leads to a 15% increase in 3-year OS (2–28%; 95% CI) [71]. However, due to the small sample size of this trial, both regimens are still recommended for cCRT.

Granulocytopenia is the most frequent hematologic adverse event in oncologic patients. It occurs in 20 to 40% of NSCLC patients under chemotherapy, as platinum-based drugs are detrimental to hematopoiesis [72]. Therefore, patients can develop some degree of immunosuppression and become especially susceptible to opportunistic bacterial and fungal infections. Further care is necessary to avoid the development of infectious diseases in the cancer population.

Thus, the treatment of choice will depend mainly on the risk of toxicity to the patient (especially neutropenia and esophagitis). Carboplatin plus paclitaxel is associated with less toxicity and is usually preferred over CE in most centers [73]. Additionally, the PROCLAIM trial demonstrated that the combination of cisplatin plus pemetrexed resulted in similar OS as cisplatin plus etoposide, with less associated adverse events in stage III NSCLC with non-squamous histology and, thus, is considered as an alternative to the regimens above [74]. Hence, the decision-making process should be performed within an MDT, considering the patient tolerance profile and physician expertise.

#### 3.3.2. Radiotherapy Component

Before determining the optimal treatment plan for an NSCLC patient, an accurate assessment of their overall fitness, medical comorbidities, cardiorespiratory reserve, genomic background, tumor stage, and mutation status is necessary. The RTOG 7301 trial analyzed 376 patients randomized into four arms of different fractionation doses and was one of the first trials to determine an optimal radiotherapy regimen for locally advanced NSCLC control. In the study, a 60 Gy regimen fractionated in 30 days (2 Gy daily fractions) led to a significantly better overall 3-year intrathoracic failure rate (33%) when compared to other regimens (41%, 44%, and 52%; *p* = 0.02). Thenceforth, the dose of 60 Gy fractionated in 30 days was established as the standard of choice radiation regimen for stage III NSCLC [75]. The RTOG 0617 phase III trial compared 544 patients undergoing cCRT submitted to either a conventional dose (60 Gy) or high dose (74 Gy) regimen in either a 3D conformal technique or intensity-modulated radiation therapy (IMRT). Doses higher than 70Gy were associated with an increased risk of death compared to conventional 60 Gy doses (median 20M vs. 29M, respectively) (HR 1.38; 95% CI: 1.09–1.76). This study was the first trial to demonstrate a better toxicity profile from IMRT. The IMRT technique was associated with lower heart doses and less grade 3 pneumonitis (7.9% vs. 3.5%; multivariate analysis OR 0.41; 95% CI: 0.17–0.99) [76].

In regard to fractionation schedules, a meta-analysis including 10 trials and a total of 2000 patients with non-metastatic NSCLC compared modified radiotherapy schedules (accelerated, hyperfractionated, or both) with conventional radiotherapy schedules. In modified schedules, the same radiation dose is administered within a hyperfractionated schedule (more fractions in a day with a lower dose per fraction), accelerated schedule (standard dose per fraction, given over a shorter period of time), or both. Thus, more evidence is necessary to address the superiority of modified fractionation schedules over the conventional, especially considering the logistical challenges for patients to attend to accelerated or hyperfractionated schedules.

A single-center experience reported that MDT discussions identified 17% of patients with large tumor volumes that precluded curative RT, a legitimate reason that pointed out the importance of MDTs in NSCLC patient management [77]. Similar to chemotherapy, the choice of radiation technique (3D or IMRT) should be made within an MDT, considering resource availability within the facility, tumor characteristics, and patient profile.

#### 3.3.3. Smoking Cessation

Continuous smoking after cancer diagnosis impacts negatively on overall survival, cancer-specific mortality, efficacy of therapy, and cancer treatment adverse events in all cancer types, including NSCLC [51]. MDT care is an excellent opportunity to address tobacco use with NSCLC patients, but this approach requires coordination into the patient treatment plan since most patients have smoked for several years and have tried to quit several times [78]. Cigarette smoking is the major risk factor for lung cancer and is estimated to account for 90 percent of all cases, with current smokers representing 38% of the lung cancer patients at diagnosis in a national survey in the United States [79]. Persistent smoking after lung cancer diagnosis impacts overall mortality, cancer-specific mortality, quality of life, efficacy of therapy, and cancer treatment toxicity [51]. A meta-analysis evaluated the influence of smoking cessation after diagnosis of early stage lung cancer and showed evidence that quitting smoking improves prognosis in this clinical setting [37].

Multidisciplinary care settings, including the easy communication among MDT members, bring unique aspects that can support the implementation of high-quality, best-in-class smoking cessation services through the use of a navigator [78].

### 3.4. Impact of MDTs on the Management of Adverse Events

Adverse events are a common feature of all known cancer therapies. In NSCLC, the most commonly reported adverse events in clinical trials and cohorts are neutropenia associated with chemotherapy, and esophagitis/pneumonitis associated with radiotherapy [6]. Hence, accurate assessment of patients and adequate management of adverse events is crucial to maintain treatment adherence and promote quality of life for patients (Supplementary Table S3).

#### 3.4.1. Pneumonitis

Pneumonitis is a common AE associated with chemoradiotherapy. The rate of grade ≥ 3 pneumonitis ranges from 5 to 10% of patients depending on the radiotherapy technique and chemotherapy agents [80]. A meta-analysis of 836 patients who underwent cCRT with cisplatin and etoposide or carboplatin and paclitaxel found out that the rate of grade ≥ 2 pneumonitis was 29.8% [81]. Pneumonitis is caused by direct cytotoxic effects, oxidative stress, and immune-mediated injury to the sensitive lung parenchyma. Radiation pneumonitis encompasses an early phase characterized by acute lung tissue inflammation as a result of exposure to radiation and a late phase that results from chronic pulmonary tissue damage and fibrosis, with potentially irreversible damage (loss of pulmonary function) and an estimated 2% risk of death [72].

#### 3.4.2. Esophagitis

Esophagitis is the most common AE reported during cCRT, as radiotherapy and chemotherapy exert independent and synergistic effects on the esophagus, a rapidly proliferating squamous epithelium. In a meta-analysis of 13 studies with stage III NSCLC patients, the mean rate of grade ≥ 3 esophagitis during cCRT with cisplatin was 18% [82]. It is a usually acute condition characterized by pseudomembranous inflammation of the esophagus whose symptoms start two or three weeks after radiotherapy. The most common symptoms of radiation esophagitis are dysphagia, odynophagia, and reflux-like symptoms such as epigastric and sternal chest pain, which may be exacerbated by preexisting esophageal disease [72]. More severe symptoms such as weight loss, esophageal obstruction, and ulceration are seen in grade ≥ 3 esophagitis. Late complications, most notably esophageal strictures, may be observed from 6 months to 5 years after cCRT [83].

Management of esophagitis is mainly symptomatic. Grade 1/2 esophagitis can be managed through the use of analgesics (such as lidocaine), proton pump inhibitors, diet modifications to avoid irritating foods, and smoking cessation. Grade ≥ 3 esophagitis may require hospitalization and more invasive intervention. A nasogastric feeding tube and surgical intervention may be necessary. These severe cases are usually characterized by varying degrees of fibrosis, leading to irreversible damage and permanent dysphagia [83].

As discussed, MDTs with nursing, nutritional, and psychological professionals can potentially better address supportive care measures and habit changes. There are clinical features associated with increased rates of acute esophagitis, such as low body mass index, gastroesophageal reflux disease, poor initial performance status, and pretreatment dysphagia [84]. Most importantly, the quantitative analysis of normal tissue effects in the clinic reports the estimated risk of acute esophagitis concerning mean organ dose to the entire esophagus. <34 Gy in 3D-radiotherapy was estimated to result in a 5–20% incidence of grade ≥ 3 esophagitis [85]. Additionally, candidiasis is common following radiation esophagitis. Patients with persistent symptoms should be started on antifungal therapy. Hence, the identification of these clinical features by MDTs is essential to carefully monitor patients with these characteristics and adopt strategies to minimize radiation delivered to the esophagus. Considering that OS in patients with advanced-stage disease worsens with cumulative intervals of delay, it is indispensable to avoid eventual interruptions of cCRT [40].

### 3.5. Immunotherapy-Based Consolidation

Immune checkpoint inhibitors are drugs that block critical proteins involved in the activation of immune cells and have demonstrated significant survival improvements in stage III NSCLC [86]. The phase III PACIFIC trial demonstrated for the first time that consolidative immunotherapy with durvalumab for patients with locally advanced unresectable stage III NSCLC without disease progression after cCRT led to significantly higher progression-free survival (PFS) and OS compared to placebo (median PFS: 16.8 months vs. 5.6 months, respectively; *p* < 0.001) [4]. The addition of immunotherapy to cCRT presents no increased risk of adverse events compared to cCRT alone [4]. Of note, the durvalumab clinical benefit was achieved without impacting patient-reported outcomes [87]. Consequently, durvalumab became the new standard for consolidation therapy for unresectable NSCLC patients whose disease has not progressed following cCRT [88]. Not typical for high-cost drugs such as durvalumab, two independent groups of researchers showed a favorable cost-effectiveness outcome for durvalumab consolidation therapy [89].

#### 3.5.1. Real-World Evidence

Although PACIFIC demonstrated a significant survival benefit with durvalumab, it was conducted in an ideal setting with strict inclusion criteria, which may not fit most patients in real-world settings. In order to be enrolled in PACIFIC, patients needed to have received a V20 of less than 35% or a mean lung dose (MLD) of less than 20 Gy. They also could not have developed grade ≥ 2 pneumonitis following cCRT and had to receive treatment up to only 42 days after completion of cCRT [4]. Indeed, prospective and retrospective real-world studies have reported that 27% to 55% of the unresectable stage III NSCLC patient population treated with durvalumab after cCRT would not have been eligible to receive consolidation immunotherapy according to the PACIFIC trial enrollment criteria, mainly due to the radiation dose criteria [90,91,92,93,94,95]. Real world studies with durvalumab are summarized in Supplementary Table S4.

Real-world data after cCRT in stage III NSCLC showed that durvalumab consolidation was associated with longer PFS. In this study, the PFS benefit was also seen in 55% of patients that did not meet the criteria of the PACIFIC trial. A German analysis from the expanded access program (EAP) showed a more advanced stage in the EAP compared to PACIFIC. EAP patients’ survival was comparable to the PACIFIC trial. Oligometastatic stage IV and patients with autoimmune disease benefited from durvalumab and did not experience an increase in toxicity [96]. Three other real-world studies also reported OS similar to the PACIFIC trial, even when patients who did not fit the trial enrollment criteria were included in the analysis [91,95,97]. Overall, the real-world patient population has more comorbidities and advanced disease. These can explain, at least in part, the higher pneumonitis rates found in some real-world studies. For instance, pneumonitis grade 3 was 14.3% in the durvalumab group vs. 2.5% in the control group [90]. Offin et al. described a 21% discontinuation rate of durvalumab due to pneumonitis grade ≥ 2 [97]. Pneumonitis in patients with stage III NSCLC has been associated with a worse prognosis following durvalumab immunotherapy [91]. Although the PACIFIC trial did not include patients who developed grade ≥ 2 pneumonitis following cCRT, they have been reported to be receiving consolidation immunotherapy in real-world settings [90].

#### 3.5.2. The Critical Role of MDTs for Implementing Immunotherapy-Based Consolidation

Historically, MDTs played an essential role in the management of stage III NSCLC, starting with the diagnosis and staging work-up and culminating with the definition of resectability and patient selection for surgical versus chemoradiation-based treatment.

Implementing durvalumab consolidation for unresectable stage III NSCLC represents a new challenge for patient care. MDT support is essential during the transition from cCRT to immunotherapy, as this process usually involves transfer from a chemotherapy and radiation oncologist specialist to an immunotherapy one. Hence, poor communication during this transition could potentially delay the start of durvalumab therapy. In this setting, navigational nurses inside MDTs could integrate communication to promote efficient communication between all physicians involved in the treatment, leading to early intervention or changes in treatment when necessary and ensuring continuity of care [6].

## 4. Mapping Review

A total of 665 papers were retrieved. After eliminating 205 duplicates, the remaining 460 titles and abstracts were screened by the Corebox Team for eligibility. Inclusion and exclusion criteria were applied after full texts were obtained from potentially relevant papers. Included articles should be in concordance with the PICO question topics (Supplementary Materials) and be either a randomized controlled trial or comparative observational study.

Additionally, topic-related keywords were google-searched along with national and international oncology society and health institution websites. References of the included papers were also verified to identify additional studies and the related articles function was used. Sixteen articles were included in the mapping review. All of the 15 (93.75%) included studies were retrospective cohorts. It is noteworthy that 6 (37.5%) studies do not clearly describe the composition or the MDT model of care. Regarding stage III NSCLC, 9 of the 16 articles evaluated somehow analyzed the impact of MDTs on the outcome of this subgroup; three of these showed better survival in stage III NSCLC treated with the MDT model [15,18,20]. The most used outcomes in the studies were: mortality/survival (68.75%), time to treatment (43.75%), and clinical pathway or guideline adherence (25%) (Supplementary Table S5).

## 5. Future Directions

Multidisciplinary team management results in better clinical and process outcomes for stage III NSCLC, with evidence of improved survival. However, the lack of clear, uniform operational criteria for MDTs is a limitation. Poorly studied important points in the literature are the minimum and ideal composition of the MDT, how to train the team, how to implement MDTs in clinical practice, and which metrics should be used to assess the program’s quality. Since there is a worldwide emerging trend towards a multidisciplinary approach in cancer care, further studies are needed to address these gaps, clarify their specific advantages, and define the resources needed for implementation.

## Figures and Tables

**Table 1:** Expert Recommendation.

Topic	Expert Recommendation	Notes
Impact of MDTs on survival	The multidisciplinary team approach is related to better processes and clinical outcomes in lung cancer patients, including time from diagnosis to treatment and survival;	-Stratified data for stage III NSCLC demonstrated a 5-year survival of 19.3% vs. 9.0% (*p* < 0.001) in MDTs participants and MDTs non-participants, respectively [18]; -MDTs had a better 1-year survival rate compared to those who were not discussed (18% vs. 8%, respectively; *p* < 0.006); -Discussion at MDTs was also associated with significantly lower postoperative advanced TNM stages. Twenty-six (15%) patients who were discussed at MDTs before surgery were affected by Stages III and IV NSCLC, as compared to forty-one (24%) patients who were not discussed (*p* = 0.041) [19]; -A Taiwan cohort analyzed 32,569 patients diagnosed with NSCLC between 2005 and 2010. The results revealed that the cox regression adjusted hazard ratio of death of MDTs participants with stage III and IV NSCLC was significantly lower than that of MDTs non-participants [20].
	In patients with stage III NSCLC, these benefits could be even more pronounced, given the heterogeneity and complexity of this specific clinical scenario;	
	All patients with lung cancer should be approached by a multidisciplinary team.	
Dietary counseling and oral nutritional supplement	All patients must undergo a nutritional assessment before treatment and receive dietary counseling;	-The clinical trials are usually heterogeneous and present low methodological quality. No specific cCRT stage III NSCLC trial was identified. A systematic review focused on nutritional and clinical outcomes during chemo(radio)therapy identified eleven studies. The authors suggest an overall benefit of nutritional therapy during chemo(radio)therapy on body weight [21].
	The use of oral nutritional supplements should be added to the diet of these patients on a prophylactic basis, as they are at high nutritional risk.	
Enteral and Parenteral nutritional	The use of enteral nutrition is recommended for patients with food acceptance below 60% of their nutritional needs and/or patients who are severely malnourished or with severe esophagitis;	-Although enteral nutrition (EN) had infrequent indications in lung cancer management (<12%), EN has a potential role in NSCLC treated with cCRT with curative intent since acute severe esophagitis can limit oral intake and impact negatively on patient outcomes. Further studies assessing the efficacy of EN in higher nutritional risk NSCLC patients are required [22]. Also here, there is a lack of well-designed clinical studies for cCRT in NSCLC [23].
	Parenteral nutrition should not be used routinely and is indicated only when oral or enteral nutrition is not possible.	
Protein supplementation	Protein supplementation must be individualized considering the patient’s nutritional status and clinical conditions and indicated for patients with malnutrition or sarcopenia.	-In sarcopenic cancer patients, it is unknown whether the recommended protein ingestion of 1.5 g/kg/day is sufficient to maintain adequate body composition. More clinical studies are needed to evaluate high protein intake and adequate amino acids composition [24]. Nonetheless, as pointed out above, it is essential to reinforce that cancer patients need a protein intake of at least 1.2–1.5 g/kg/day, almost twice as many as healthy individuals (0.8 g/kg/day) [23]. Additionally, protein from animal origin should be chosen over other options of protein as they demonstrate a higher anabolic potential [25].
Omega-3 fatty acids supplementation	Should be performed in patients undergoing systemic treatment with an offer of 2 g/day.	-In a double-blind, randomized controlled trial (RCT), forty patients with stage III NSCLC undergoing cCRT were assigned to receive two cans/d of protein- and energy-dense oral nutritional supplements containing omega-3 (2.0 g EPA + 0.9 g DHA/d) or an isocaloric control supplement The omega-3 group had better weight control than the control group. The omega-3 group had better weight maintenance than the control group after week 4 (1.7 kg; *p* < 0.05), a better fat free mass after week 5 (1.9 kg; *p* < 0.05) and a reduced resting energy expenditure after week 3 [26]. Also, other results suggest that omega-3 groups improved the quality of life, performance status and physical activity [27].
Physical activity	Aerobic and resistance exercises are indicated for stage III NSCLC patients during chemotherapy, whenever possible.	-Significantly few studies have addressed exercise in NSCLC patients treated with cCRT. A meta-analysis, including a total of 2643 NSCLC patients reported that sarcopenia is an independent risk factor for postoperative death and postoperative complications [28]. A retrospective study presented sarcopenia as an independent poor prognosis factor after cCRT in stage III NSCLC [29].
Navigating nursing	The implementation of the navigation program is recommended inside the MDTs programs.	A single-center study with 408 stage IIIB/IV NSCLC patients compared care before and after the implementation of a navigational nurse program. After implementation, rates of molecular epidermal growth factor (EGFR) testing increased from 62% to 91% (*p* < 0.001); Time from patient referral to the institution to the start of radiotherapy decreased from 18 to 11.5 days, (*p* < 0.001). Most notably, significantly more EGFR molecular results were available at the time of the first medical oncologist consultation (37 after implementation vs. 6 before implementation; *p* < 0.001) [30];
	Advanced practice nurses should perform the navigation of NSCLC patients in order to provide the most benefit throughout the treatment journey.	A meta-analysis of 52 studies about cancer care coordination programs, in which nurse navigation was the most frequent approach, revealed improvements in patient experience with care [31].
Nursing care	Nurses should be trained to keep track of their patients and detect early signs of adverse events or changes in behavior, contributing to treatment safety and adherence, quality of life and satisfaction.	Nurse practitioners can observe the respiratory pattern and evaluate the need for oxygen therapy, identify the presence of cyanosis, skin pallor and dyspnea [32].
Impact of MDTs on cCRT	-Treatment decisions for optimal multimodality treatment in patients with stage III NSCLC should be discussed in a MDT approach, including fitness for concurrent versus sequential treatment and the choice of radiation and chemotherapy regimens; -An accurate assessment of their overall fitness, medical comorbidities, cardiorespiratory reserve, genomic background, tumor stage and mutation status is important prior to the treatment plan definition; -A IMRT 60 Gy regimen fractionated in 30 days (2 Gy daily fractions) is the optimal radiotherapy regimen for locally advanced NSCLC for most cases; -Modified schedules (accelerated and/or hyperfractionated) should only be used in patients with a good performance status (0 or 1) and in low risk for toxicity (see adverse events session); -Platin based combinations, including carboplatin and paclitaxel, cisplatin and etoposide and cisplatin and pemetrexed (for non-squamous histology) are considered equally effective in this scenario, and the choice of regimen is based in patients characteristics and expertise of the center.	-An analysis of the impact of MDT meetings on the management of 55 lung cancer patients revealed that MDT meetings changed management plans in 58% of cases (95% CI: 45–71%; *p* < 0.005). These changes were characterized by additional investigations (59%), or changes in treatment modality (19%), treatment intent (9%), histology (6%), or tumor stage (6%), and the meeting recommendations were implemented in 72% of cases [33]; -A meta-analysis results demonstrated that a modified schedule improved overall survival (HR 0.88; 96% CI 0.80 to 0.97, *p* = 0.009) and resulted in a 5-year survival rate benefit of 2.5% (8.3% to 10.8%). However, modified schedules were associated with a significantly increased risk of acute esophageal toxicity [34].
Smoking cessation	The multidisciplinary care settings, including the ease of communicating among MDT members, bring unique aspects that can support the implementation of high-quality, best-in-class smoking cessation services through the use of a navigator.	-Smoking during radiotherapy led to worse locoregional control for NSCLC [35] and continuing cigarette smoking by patients receiving cCRT in limited-stage small-cell lung cancer was associated with decreased survival (13.6 vs. 18 months) compared with former smokers [36]. In early NSCLC, continued smoking was associated with a significantly increased risk of all-cause mortality and recurrence [37]. Non Smoking status significantly predicts more prolonged overall survival (OS) in stage III NSCLC in a real-world study [38].
The critical role of MDTs for implementing immunotherapy-based consolidation	-Multidisciplinary approach is essential in stage III NSCLC, in order to better select treatment strategy as well as to follow up patients undergoing CRT and durvalumab; -Careful multidisciplinary monitoring is necessary for patients who start on immune-checkpoint inhibitors with a history of radiation pneumonitis during cCRT in order to optimize supportive care, patient education on pulmonary symptoms, and early diagnosis and intervention; -In the PACIFIC trial, early initiation of durvalumab after concomitant CRT (≤14 d) was associated with a trend towards higher OS. Chest CT should be performed as soon as possible after completion of RT (preferably <4 wk): importantly, the role of chest CT is not to assess response, but rather to rule out local progression and/or signs of severe pneumonitis, which would contraindicate consolidation durvalumab therapy [39]; -If immunotherapy has to be interrupted due to pneumonitis, the decision about an eventual re-challenge should be made within MDTs [40].	-PFS and OS benefit with durvalumab was described across all PD-L1 subgroups [41]. The 5-year OS rate for durvalumab was 42.9% against 33.4% for placebo [42]; -The higher pneumonitis incidence in real-world studies should be a red flag to health care professionals, and it can also be an opportunity to involve structured MDTs care to minimize the impact of pneumonitis in clinical practice. Thus, MDTs play a crucial role in daily practice to guarantee high-quality care and benefit patients’ clinical outcomes [39].

## Supplementary Materials

The following supporting information can be downloaded at: https://www.mdpi.com/article/10.3390/curroncol31010012/s1, Methodology S1. Mapping review; Figure S1. PRISMA flow chart of search strategy divided by identification, screening, eligibility, and inclusion; Methodology S2. Search strategy; Table S1. Nutritional and physical activities interventions [24,46,50,98,99,100,101,102,103,104,105,106,107,108,109,110,111,112,113,114]; Table S2. Recommended cCRT Regimens [115]; Table S3. MDTs and Adverse Events in stage III NSCLC [40,51,58,72,84,85,116,117]; Table S4. Real World Studies with Durvalumab; Table S5. Mapping review results overview [10,16,18,20,62,63,118,119,120,121,122,123,124,125,126,127].
