# Supplementary material for: The Best Supportive Care in Stage III Non-Small-Cell Lung Cancer
Source: Curr Oncol. 2023 Dec 29;31(1):183–202. doi: 10.3390/curroncol31010012 (PMC10814676; doi:10.3390/curroncol31010012)
Supplement: Supplementary file 1 [file curroncol-31-00012-s001.zip › curroncol-2747386-supplementary.pdf]

Supplementary Materials

Methodology S1.Mapping review

The mapping review can map out and categorize existing literature and identify gaps from which further primary research can be planned to fill.

PICO - Multidisciplinary team (MDT) overall role

- **PICO question:** In patients with unresectable stage III NSCLC who receive chemoradiation treatment, does management with a multidisciplinary team improve outcomes?
    - **P:** NSCLC stage III receiving chemoradiation
    - **I:** MDT
    - **C:** no MDT
    - **O:** death, progression-free survival, time to progression, quality of life, chemoradiation complications, chemotherapy-related complications, chemotherapy deaths, radiotherapy-related complications, radiotherapy-related deaths, severe adverse events, adverse events, pneumonitis, esophagitis, neutropenia, immunotherapy treatment, duration of chemotherapy, duration of radiotherapy, dose of chemotherapy, dose of radiotherapy, dose of immunotherapy, patient management, EAs surveillance.

The literature review was reported according to the AMSTAR and the 2009 Preferred Reporting Items for Systematic Reviews and Meta-Analysis (PRISMA) guidelines.

A total of 760 papers were retrieved. After eliminating 259 duplicates, the remaining 501 titles and abstracts were independently screened by two authors for eligibility. Inclusion and exclusion criteria were applied after full texts were obtained from potentially relevant papers. Included articles should be in concordance with the PICO question topics (Supplement 1) and be either a systematic review, meta-analysis, randomized controlled trial, or comparative observational study.
Additionally, topic-related keywords were Google-searched along with national and international Oncology Societies and Health Institutions websites. References of the included papers were also verified to identify additional studies, and the related articles function was used. In total, 16 articles were included in this literature review.

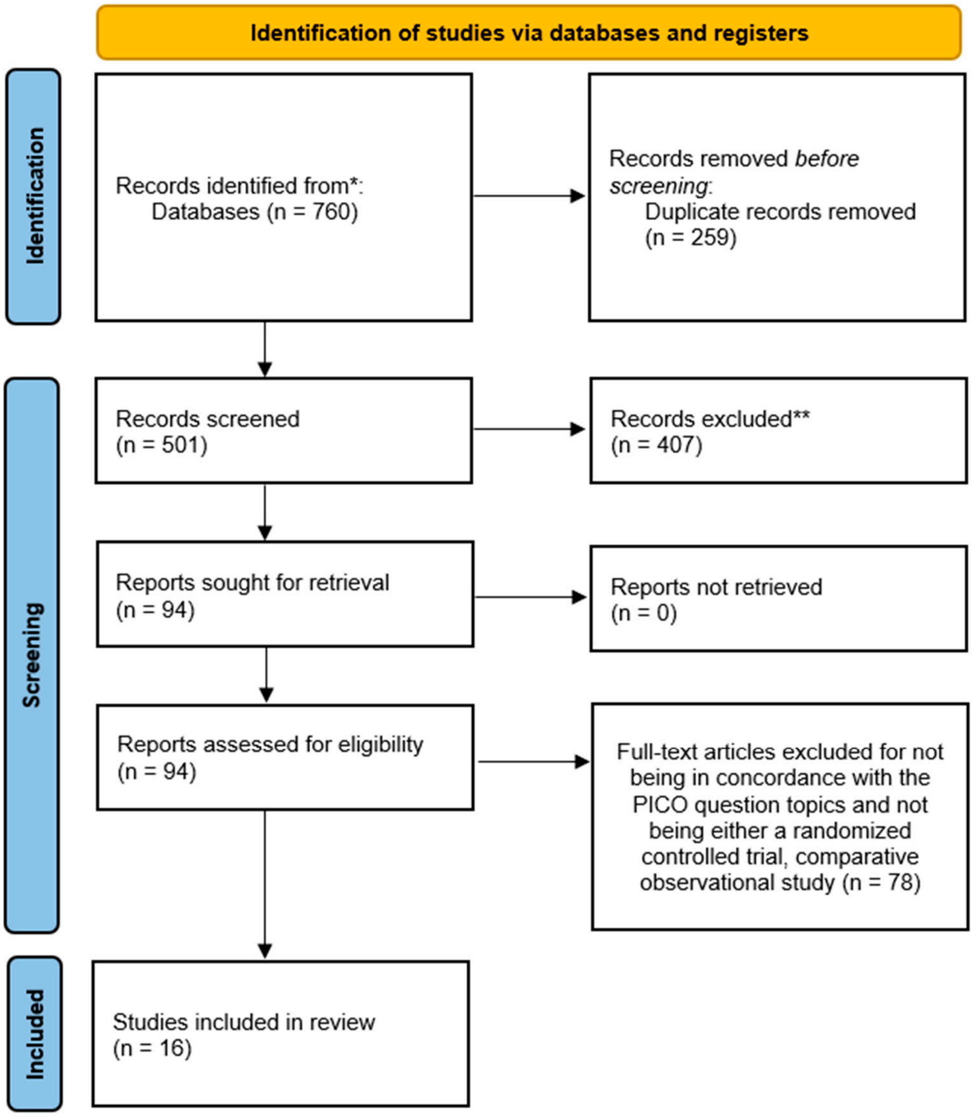

Figure S1. PRISMA flow chart of search strategy divided by identification, screening, eligibility, and inclusion.

Methodology S2.Search strategy

Databases: Cochrane Library, EMBASE, Lilacs, Pubmed, Web of Science
Search date: May 18th, 2023
Total: 760
Duplicates: 259
After removing duplicates, 501

Cochrane - 20
#**1** (chemoradiotherapy OR chemoradiation):ti,ab,kw - 9358
#**2** MeSH descriptor: [Chemoradiotherapy] explode all trees - 2204

#**3** #1 OR #2 - 9358
#**4** "Non-Small Cell Lung Carcinoma" OR "Nonsmall Cell Lung Cancer" OR "Nonsmall Cell Lung Cancer" : ti,ab,kw - 14613
#**5** "carcinoma, nonsmall cell lung"[MeSH Terms] -5734
#**6** #4 OR #5 - 15333
#**7** Multidisciplinary OR Interdisciplinary OR Transdisciplinary :ti,ab,kw - 10346
#**8** "interdisciplinary studies"[MeSH Terms] - 19
#**9** #7 OR #8 - 10347
#**10** #3 AND #6 AND #9 - 20

EMBASE - 522
('non small cell lung cancer'/exp OR 'carcinoma, non-small-cell lung':ti,ab,kw OR 'lung cancer, non small cell':ti,ab,kw OR 'lung non small cell cancer':ti,ab,kw OR 'lung non small cell carcinoma':ti,ab,kw OR 'non small cell lung cancer':ti,ab,kw OR 'non small cell lung carcinoma':ti,ab,kw OR 'non-small-cell lung carcinoma':ti,ab,kw OR 'non small cell cancer, lung':ti,ab,kw) AND ('chemoradiotherapy'/exp OR 'chemoradiation':ti,ab,kw OR 'chemoradiotherapy':ti,ab,kw OR 'radiochemotherapy':ti,ab,kw) AND ('multidisciplinary team'/exp OR 'multi-disciplinary team' OR 'multidisciplinary team' OR multidisciplinary OR transdisciplinary OR interdisciplinary)

#1 'chemoradiotherapy'/exp OR 'chemoradiation':ti,ab,kw OR 'chemoradiotherapy':ti,ab,kw OR 'radiochemotherapy':ti,ab,kw - 102.545
#2 'non small cell lung cancer'/exp OR 'carcinoma, non-small-cell lung':ti,ab,kw OR 'lung cancer, non small cell':ti,ab,kw OR 'lung non small cell cancer':ti,ab,kw OR 'lung non small cell carcinoma':ti,ab,kw OR 'non small cell lung cancer':ti,ab,kw OR 'non small cell lung carcinoma':ti,ab,kw OR 'non-small-cell lung carcinoma':ti,ab,kw OR 'non small cell cancer, lung':ti,ab,kw - 218.183
#3 'multidisciplinary team'/exp OR 'multi-disciplinary team' OR 'multidisciplinary team' OR multidisciplinary OR transdisciplinary OR interdisciplinary - 424.736
#4 #1 AND #2 AND #3 - 522

Lilacs - 0 (recovered only from Medline)

(chemoradiotherapy OR chemoradiation OR quimiorradiação OR quimiorradioterapia) AND ("Non Small Cell Lung Carcinoma" OR "Nonsmall Cell Lung Cancer" OR "Non small Cell Lung Cancer" OR "Carcinoma Pulmonar de não Pequenas Células" OR "Carcinoma de Pulmão de Células não Pequenas" OR "Carcinoma Pulmonar de Células não Pequenas" OR "Câncer Pulmonar de Células não Pequenas" OR "Carcinoma de Pulmão de não Pequenas Células") AND (Multidisciplinary OR Interdisciplinary OR Transdisciplinary OR multidisciplinar OR interdisciplinar OR transdisciplinar)

Pubmed - **128**
("chemoradiotherapy"[Title/Abstract] OR "chemoradiation"[Title/Abstract] OR "chemoradiotherapy"[MeSH Terms]) AND ("Non Small Cell Lung Carcinoma"[Title/Abstract] OR "Nonsmall Cell Lung Cancer"[Title/Abstract] OR "Non small Cell Lung Cancer"[Title/Abstract] OR "carcinoma, non small cell lung"[MeSH

Terms]) AND ("Multidisciplinary"[All Fields] OR "Interdisciplinary"[All Fields] OR "Transdisciplinary"[All Fields] OR "interdisciplinary studies"[MeSH Terms])

#**1** "chemoradiotherapy"[Title/Abstract] OR "chemoradiation"[Title/Abstract] OR "chemoradiotherapy"[MeSH Terms] - 46,498
#**2** "Non Small Cell Lung Carcinoma"[Title/Abstract] OR "Nonsmall Cell Lung Cancer"[Title/Abstract] OR "Non small Cell Lung Cancer"[Title/Abstract] OR "carcinoma, non small cell lung"[MeSH Terms] -96,498
#**3** "Multidisciplinary"[All Fields] OR "Interdisciplinary"[All Fields] OR "Transdisciplinary"[All Fields] OR "interdisciplinary studies"[MeSH Terms] - 282,944

#**4** #1 AND #2 AND #3

Web of Science - 90

#1 TS=(chemoradiotherapy OR chemoradiation) - 53.173
#2 TS=("Non Small Cell Lung Carcinoma" OR "Nonsmall Cell Lung Cancer" OR "Non small Cell Lung Cancer") - 102.930
#3 TS=(Multidisciplinary OR Interdisciplinary OR Transdisciplinary) - 238.730
#4 #1 AND #2 AND #3 109

Table S1.Nutritional and physical activity interventions

Dietary counseling and oral nutritional supplement	- Disease-specific ONS have a macro-and micronutrient composition adapted to the predicted needs of a specific disease like lung cancer [98]. ONS improves muscle mass and has a positive impact in cancer patient sarcopenia [50]. A randomized controlled trial (RCT) in head and neck cancer patients undergoing radiotherapy reported that ONS reduced 60% the need for changes in scheduled anti-cancer therapy [99]. A meta-analysis among malnourished medical inpatients or patients at nutritional risk reported that patients receiving nutritional support improved survival and nonelective hospital readmission rate [100];
Enteral and Parenteral nutritional	-. Many guidelines recommend the enteral route to be the first choice if oral nutrition remains inadequate after optimal intervention (DC and ONS) [46];
Protein supplementation	- Protein intake can be low, even in obese or overweight oncologic patients. A study revealed that 35% of obese or overweight patients with lung and colorectal cancer consumed less than 1.0g/kg of daily protein. A cohort study in patients with stage III colon cancer showed that a higher animal protein intake is associated with lower 7-year mortality [101]. These findings suggest a benefit from a higher protein intake in the oncologic population;<br>- Whey protein supplementation as a nutritional intervention to reach the recommended protein intake during cancer treatment has attracted increasing attention. A single-center randomized controlled trial with 166 malnourished advanced cancer patients with mixed cancer types demonstrated that whey protein isolate supplementation (20g/day) during three months resulted in improved body weight, muscle strength, and reduced risk of chemotherapy toxicities. The reduction was stronger for severe (grade>3) adverse events [102]. Despite the above-mentioned study, most literature assessing protein and amino acid intake have focused on metabolic endpoints (e.g. protein synthesis) instead of clinical endpoints [103];
Omega-3 fatty acids supplementation	- EPA and DHA are essential nutrients for humans [104]. EPA/DHA reduces inflammation, promotes weight gain and nutrient intake, and improves performance status in non-randomized studies [24];<br>- A systematic review for chemotherapy and/ or radiotherapy patients selected ten high-quality studies over 157 and found

	that combining omega-3 fatty acids supplements with conventional chemotherapy was beneficial. The authors pointed out that none of the studies reported a worse clinical endpoint for the supplement group [105];<br>- In NSCLC, a clinical study (n=40) evaluated nutritional intervention with fish oil (2.2g EPA/day) over the standard of care (SOC) in chemotherapy patients. During chemotherapy, 69% of patients in the EPA arm maintained or gained muscle versus 29% in the SOC arm. Higher plasma EPA concentration was associated with the most significant muscle gain[106].<br>- Another RCT with ninety two patients with advanced NSCLC receiving chemotherapy evaluated diet plus ONS containing EPA (ONS-EPA) vs. only isocaloric diet (C). ONS-EPA arm had significantly greater energy and protein intake compared with control. Neuropathy, fatigue and anorexia were also improved in the ONS-EPA arm [107];
Physical activity	- Exercise training during cancer therapy improves physical function, reduces cancer-related fatigue (CRF) and increases symptom control and quality of life [108]. A large meta-analysis (113 studies and 11,525 patients) has demonstrated that exercise is more effective to improve CRF than pharmacological approach [109];<br>- A Cochrane review in advanced lung cancer demonstrated that exercise capacity was significantly higher in the interventional group versus control group [110];<br>-In a RCT, the researchers investigated the effect of curative intent therapy (surgery, RT, or CRT) and two rehabilitation programs for stage I-III lung cancer. The primary endpoint was a change in 6-min walking distance (6MWD) after rehabilitation. The study showed that curative intent therapy significantly impaired patients' exercise capacity. Resistance training significantly improved and restored functional exercise capacity [111];<br>- A relatively new area of study is the role of exercise in prehabilitation. A recent meta-analysis suggested that prehabilitation can reduce hospital stay in cancer patients undergoing major abdominal surgery. Although no studies address prehabilitation in NSCLC treated with cCRT, this can be a helpful approach in this clinical scenario. Further studies are needed to evaluate these benefits [112];<br>- A meta-analysis with meta-regression has shown that protein supplementation can positively impact muscle size and strength during resistance exercise training in healthy adults [113];<br>- In a double-blind, placebo-controlled RCT, essential amino acids (EAA) combined with aerobic exercise for 24 weeks improved muscle strength and muscle protein synthesis compared with aerobic exercise or EAA alone [114]. A large RCT is currently ongoing and will evaluate the impact of physical activity, nutritional counseling, and dietary intervention in patients

	with sarcopenia [50];

Table S2. Recommended cCRT Regimens

Recommended cCRT Regimens[115]	
Chemotherapy Regimens§	Radiotherapy Regimens
Carboplatin AUC 5 on day 1, pemetrexed 500 mg/m^2 on day 1 every 21 days for 4 cycles	Thoracic RT, 60-70 Gy
Cisplatin 75mg/m2 on day 1, pemetrexed 500 mg/m^2 on day 1 every 21 days for 3 cycles + pemetrexed 500 mg/m2 for 4 cycles	Thoracic RT, 60-70 Gy
Paclitaxel 45-50 mg/m^2 weekly, carboplatin AUC 2 + additional 2 cycles every 21 days of paclitaxel 200 mg/m^2 and carboplatin AUC 6	Thoracic RT, 60-70 Gy
Cisplatin 50 mg/m^2 on days 1, 8, 29, and 36, etoposide 50 mg/m2 days 1-5 and 29-33	Thoracic RT, 60-70 Gy

§ When consolidation immunotherapy (durvalumab) is used after definitive cCRT, additional chemotherapy after radiation is not recommended.

Table S3. MDTs and Adverse Events in stage III NSCLC

Adverse event	MDT role
Cardiac toxicity	- MDTs could impact the management of patients with a history of cardiac problems by choosing the optimal method of radiation delivery in order to minimize cardiac risks, potentially contributing to more patients fit for cCRT. The diagnostic and treatment of RT cardiac injury are similar to other cardiac patients [51]. Cardiac toxicity is a concern during chest radiotherapy when high doses of radiation are delivered to the heart [116].
Granulocytopenia	- MDT close monitoring is required to avoid the development of serious infectious diseases in these patients. Granulocytopenia is the most frequent hematologic adverse event in oncologic patients. It occurs in 20 to 40% of NSCLC patients under chemotherapy, as platinum-based combinations are detrimental to hematopoiesis [72].
Pneumonitis	- MDTs interventions can avoid further complications that could impair quality of life and/or lead to interruption of cCRT. Investigation and monitoring by an MDT consisting of navigational and practitioner nurses, radiologists, radiation oncologists, pneumologists and medical oncologists are essential for confirming the diagnosis and identifying the pneumonitis etiology, as well as determining the optimal treatment and management and eventual adoption of alternative radiotherapy techniques with lesser irradiation of healthy tissues and chemotherapy regimen;<br>- Pneumonitis demands early intervention to minimize risks of serious complications, so input from MDTs is fundamental for the differential

	diagnosis from infectious pneumonia, chronic obstructive pulmonary disease exacerbation, and tumor progression or the mass effect of the primary neoplasm on airways. In this scenario, nurse practitioners who are in constant contact with patients play a vital role in the early detection of symptoms [58]. Pneumonitis displays hallmarks of acute inflammation around one month before the onset of respiratory and fibrosis symptoms (nonproductive cough, presence or worsening of dyspnea, chest pain) that can be monitored, such as increased serum interleukin-6, interleukin-1α, and tumor necrosis factor-α [117];
Esophagitis	- MDTs with nursery, nutrition and psychology professionals can potentially better address supportive care measures and habit changes. There are clinical features associated with increased rates of acute esophagitis, such as low body mass index, gastroesophageal reflux disease, poor initial performance status, and pretreatment dysphagia [84]. Most importantly, the Quantitative Analyses of Normal Tissue Effects in the Clinic report estimated the risk of acute esophagitis concerning mean organ dose to the entire esophagus. $<$34 Gy in 3D-radiotherapy was estimated to result in a 5%–20% incidence of grade $\geq$ 3 esophagitis [85].<br>- The identification of these clinical features by MDTs is essential to carefully monitor patients with these characteristics and adopt strategies to minimize radiation delivered to the esophagus. Considering that OS in patients with advanced-stage disease worsens with cumulative intervals of delay, it is indispensable to avoid eventual interruptions of cCRT [40];

Table S4. Real World Studies with Durvalumab

Study	Year	N	% of patients ineligible to PACIFIC	PFS overall population	PFS subgroup ineligible to PACIFIC	OS overall population	Pneumonitis	Country
Grivet	2019	121	31%	not reported	not reported	not reported	not reported	Brazil
Barbaro	2019	146	27%	not reported	not reported	15 mo OS rates 100% vs 85%	not reported	US
Sakaguchi	2019	81	30%	not reported	not reported	not reported	≥ Grade 2 was 16.4%	Japan
Jung	2020	61	55%	not reported vs. 9.6 mo	not reported vs. 6.4 mo	not reported	Grade 3 was 14.3% vs 2.5%	Korea
Eichkorn	2020	437	50%	not reported	not reported	not reported	not reported	Germany
Faehling	2020	126	not reported	24 mo PFS of 46.7%	20.1 (autoimmune disease) and 13.3 mo (stage IV)	24 mo PFS of 66%	> Grade 3 was 8.7%	Germany

Desilets	2021	147	32%	not reported	not reported	not reached vs. 26.9 mo	≥ Grade 3 was 6.1%	Canada/Japan

Table S5. Mapping review results overview

Author, year	Study design	N	MDT model and/or team members	Stage III analysis	Outcomes	Notes
Murray, 2003 [118] *	RCT	88	Not clearly specified (multidisciplinary team).	No	(i) 4-week time to first treatment.<br>(ii) % of radical treatment rate	Conventional chest clinic vs centralized two-stop pathway. It´s a feasibility study. The results show several advantages to investigations and diagnosis in the central arm, particularly in time to treatment initiation, patient satisfaction and rate of radical treatments;
Riedel, 2006 [119]	Cohort	345	Pulmonologist organized the clinic, but multidisciplinary discussions occurred before and during a patient's visit among specialists from medical oncology, radiation oncology, and pulmonary medicine. TMC: thoracic malignancy care conference.	No	(i) time from initial presentation to diagnosis<br>(ii) time from diagnosis to treatment initiation<br>The study failed to reveal the benefit of MDTs vs no MDTs care model.	Potential confounders include the absence of a surgeon in the TMC setting, an ongoing weekly multidisciplinary conference in the non-TMC cohort, and existing infrastructures based on previous TMC experiences and past provider experience;
Bydder, 2009 [120]*	Cohort	98, MDT (81) and	A weekly MDT lung cancer meeting was held at the hospital. The MDT consisted of respiratory physicians, cardiothoracic surgeons, medical oncologists, a radiation	No	(i) mean survival: 280 vs. 205 days (p=0,048)	Few patients in the non-MDT cohort and non-MDT patients with ≥ stage IV (82% vs. 57%);

		non MDT (17)	oncologist, a palliative care physician, a radiologist, a pathologist, a nuclear physician, and a specialist lung cancer nurse, as well as doctors receiving specialist training. Discussion typically included review of the case history, review of both imaging studies and histopathological findings, followed by MDT commentary. Patient details and the outcome of MDT discussion, including provisional management plans, were documented on a prospective database accessible through the hospital intranet and in a hardcopy summary retained in the patients' medical records.			
Freeman, 22010 [121]*	Cohort	1222, 535 no MDT and 687 MDT	At minimum, an evaluation by a thoracic surgeon, pulmonologist, and medical oncologist for patients with a clinical stage I or II malignancy or evaluation by the above as well as radiation oncology for stage III or IV patients.	No	(i) complete staging evaluation;<br><br>(ii) multidisciplinary evaluation prior to therapy;<br><br>(iii) adherence to NCCN guidelines<br><br>(iv) mean days from diagnosis to treatment	The number of patients receiving a complete staging evaluation (79%/93%: $p < 0.0001$), multidisciplinary evaluation prior to therapy (62%/96%: $p < 0.0001$) and adherence to the National Comprehensive Cancer Network (NCCN) treatment guidelines (81%/97%: $p < 0.0001$) all increased significantly while mean days from diagnosis to treatment significantly decreased (29/17: $p < 0.0001$) following the initiation of a TMC;
Boxer, 2011 [10]*	Cohort	988, 504 MDT and 484 no MDT	Not reported	Yes. Logistic Regression Model of Factors That Predicted	(i) Treatment receipt.<br><br>(ii) survival	The median patient age was 69 years and 73 years in the MDT group and the non-MDT group, respectively ($P < .01$). There was no pathologic diagnosis for 13% of non-MDT patients compared with 4% of MDT patients ($P < .01$). Treatment receipt for MDT patients versus non-MDT patients was 12% versus 13%, respectively, for surgery (P

				for Receipt of Treatment		value nonsignificant); 66% versus 33%, respectively, for radiotherapy (P < .001); 46% versus 29%, respectively, for chemotherapy (P < .001); and 66% versus 53%, respectively, for palliative care (P < .001). In patients with good performance status, the MDT group had significantly better receipt of radiotherapy among patients with stage I through IV NSCLC and had significantly better receipt of chemotherapy among patients with stage IV NSCLC. MDT discussion was an independent predictor of receiving radiotherapy, chemotherapy, and referral to palliative care but did not influence survival;
Pan, 2015 [20]*	Cohort	32,569	Not clearly reported.	Yes	Multivariate Cox proportional hazards model was used to explore whether the involvement of MDT care had an effect on survival. This study applied the propensity score as a control variable to reduce selection bias between patients with and without involvement of MDT care.	The adjusted hazard ratio (HR) of death of MDT participants with stage III & IV NSCLC was significantly lower than that of non-MDT participants (adjusted HR = 0.87, 95% confidence interval = 0.84-0.90). This study revealed that MDT care is significantly associated with higher survival rate in patients with stage III and IV NSCLC and, thus, MDT care should be used in the treatment of these patients;
Senter, 2016 [63]	Cohort	308, MDC (139) and non-MDC (169)	All day consultation clinic with the following elements: (1) co-localization of specialists who see patients in an integrated, shared clinic space, (2) one on-one tumor board style discussion of each patient prior to patient counseling, and (3)	No	(i)median number of days from initial clinical visit to first treatment:	Multivariate analysis showed that participation in MDC was an independent predictor for all-cause mortality. The MDC cohort had more T4 disease (36.7% vs. 18.9%, P < 0.01) and adenocarcinoma histology (59.7% vs. 45.2%, P = 0.024);

			combined patient/family education covering nutrition, palliative and hospice care, clinical research, and social work resources.		24 vs 33 days (p<0.01)<br>(ii) time to start cCRT: 89.4% vs. 62.6% started within 7 days (p<0.01);<br>(iii) survival time: 30.9 vs. 21.5 months (p<0.01)	
Rogers, 2017 [122]*	Cohort	593, 294 MDM and 299 no MDM	MDM has a defined participant list with a quorum of treating physicians consisting of at least one surgeon, medical oncologist, radiation oncologist, pathologist, and respiratory physician. It also has allied health, supportive care staff and other relevant staff attending, as well as family physicians of their representatives.	No	(i) mortality	Lung cancer patients that were presented to a MDM prior to treatment had a significant reduction in mortality (lung cancer hazard ratio [HR] 0.62, 95% confidence interval [CI] 0.50e0.76, P < 0.01);<br>MDM after adjusting for the potential cofounders of age, stage, comorbidities, and treatment;<br>This cohort also evaluated breast, hematological and colorectal cancer;
Bilfinger, 2018 [18]*	Cohort	1956 MDT and 2315 standard care	Thoracic surgery, interventional pulmonology, medical oncology, radiation oncology, and 2 dedicated nurse practitioners as the core group. Interventional radiology, radiation therapy, chest radiology, and social and nutritional support are also on site.	Yes	(i) survival	The 5-year survival rates in the propensity-matched sample were one third greater among MDT patients compared with those receiving a traditional care approach (33.6% vs. 23.0%; P < .001). After adjusting for potential confounders in the multivariable propensity-matched analyses, the MDT model demonstrated a significant beneficial effect on 5-year survival outcomes compared with the standard treatment model (hazard ratio, 0.65; 95% confidence interval, 0.54-0.77);<br>Stage 3 survival - 1-y (66.6 vs 49.7; P<0.001); 3-y (30.1 vs 17.5; P<0.001); 1-y (19.3 vs 9.0; P<0.001); 10-y (7.4 vs 6.2; P<0.055);
Stone, 2018 [123]*	Cohort	1197 cases were included,	The St Vincent's Lung Cancer MDT meeting was established in 2006 and is attended by staff from a full range of	Yes	(i) The primary aim was to assess the impact of MDT	Survival analyzed by stage was greater in the MDT group at 1, 2 and 5 years for all stages except stage IIIB at 1-year post-diagnosis. Adjusted survival analysis for the entire cohort showed improved

		295 (24.6%) with MDT presentation and 902 (75.4%) without.	medical subspecialties, nursing, and allied health professionals. History and clinical progress are reviewed and management recommendations are recorded with the responsibility of implementation given to the referring clinician.		on survival after a diagnosis of lung cancer	survival at 5 years for the MDT group (HR 0.7 (0.58-0.85), p < 0.001). Survival probability at 1, 2, and 5 years by stage and whether presented at MDT:<br>- Stage IIIA – 1-y, 0.76 (0.59-0.87) vs 0.71 (0.57-0.82); 2-y, 0.66 (0.48-0.78) vs 0.37 (0.24-0.50); 5-y, 0.61 (0.43-0.74) vs 0.27 (0.15-0.39);<br>- Stage IIIB - 1-y, 0.65 (0.44-0.80) vs 0.62 (0.48-0.74); 2-y, 0.46 (0.27-0.64) vs 0.40 (0.27-0.52); 5-y, 0.38 (0.20-0.56) vs 0.23 (0.13-0.35);
Friedman, 2013 [124]	Cohort	78, TMDC (34) and non-TMDC (44)	No information	Yes	(i) adherence to clinical pathways: 84 vs.46% (p<0.001)<br>(ii) number of days from initial clinical visit to first treatment 20.62 vs. 29.03 (p not reported)	
Freeman, 2015 [16]*	Cohort	13,254 , MDC (6,627) and non-MDC (6,627)	At a minimum, patients were evaluated by a thoracic surgeon, radiation oncologist, and medical oncologist before initiation of nonemergency therapy.	No	(i) adherence to NCCN guidelines: 88 vs. 71% (p<0.0001)<br>(ii) Complete staging: 91 vs. 67% (p<0.0001)<br>(iii) Diagnosis to treatment 19±8 vs	MDC care was less expensive than non-MDC.;<br>The groups were propensity matched. Patient demographics and Charlson comorbidity scores were comparable after matching;

					32±11 days (p<0.001)<br><br>(iv) mean cost of assistance, diagnosis and staging: USD 7,212 vs 10,213 (p<0.001)	
Friedman, 2016 [62]*	Cohort	109,MDC (52) and non-MDC (57)	The thoracic MDC at LVHN meets weekly for prospective case reviews. Physicians from thoracic surgery, medical oncology, radiation oncology, diagnostic radiology, and pulmonary medicine attend to the clinic. Representatives from palliative medicine and nutrition also attend to the clinic.<br><br>Prior to each meeting, a nurse navigator reviews all cases and collects pertinent clinical data, including a full history, radiology studies, and pathology (both reviewed internally) for presentation to the group. After group discussion, if necessary, the nurse navigator will schedule tests before treatment recommendations are made. If no additional testing is needed, the team formulates a coordinated treatment plan.. If additional referrals are needed (smoking cessation, pulmonary rehabilitation, and pain management), they are made by the nurse navigator.	Yes	(i) number of days from initial clinical visit to first treatment: 19.85±13.8 vs. 29.09±27.3 (p=0.043)<br><br>(ii) adherence to stage III clinical pathway: 88.5 vs. 35.1% (p<0.001)<br><br>(iii) proportion of patients with mediastinal pathological staging: 57.7 vs. 24.5% (p<0.001)<br><br>(iv) overall survival time: 17 vs. 14 months (p=0.054)	

Harbegue, 2019 [125]	Cohort	88	No information	Yes	(i) median OS in MDT treated patients: 49 vs. 22 months ($p<0.01$)	Low quality study. The majority of the results are descriptive data of patients seen by an MDT. The only comparative result does not detail which is the comparison group;
Hung, 2020 [126]*	Cohort	515, MDT (242) and non-MDT (273)	The members of thoracic oncology MDT included chest physicians, surgeons, medical oncologists, radiation oncologists, radiologists, nuclear medicine physicians, pathologists, nurses, psychologists, and dietitians. All the specialists met once a week to discuss cases of lung cancer, especially in patients whose condition were complicated or in those with stage III tumor.	Yes	(i) median OS in MDT vs non-MDT: 39.6 vs. 25.7 months ($p=0.018$)	Cox-regression analysis was used for multivariate OS analysis;<br><br>Authors do not report the proportion of patients with stage IIIA or IIIB in the MDT and non-MDT groups;
Hergebue, 2021 [127]*	Cohort	88 patients (MDT and non-MDT)	No information	Yes	(i) independent prognostic factors influencing OS: upfront surgery + adjuvant therapy (HR 0.61; 95% CI 0.38-0.96; $p=0.034$), adherence to MDT decision (HR 0.26; 95% CI 0.15-0.47; $p<0.01$) and tumor size >7 cm (HR 2.31; 95%CI 1.29-4.13; $p=0.005$)	Multivariate analysis was performed to identify independent factor associated with survival;

* Full text article; RCT: randomized clinical trial; MDM: multidisciplinary meeting;

Supplementary References

10. Boxer, M.M.; Vinod, S.K.; Shafiq, J.; Duggan, K.J. Do Multidisciplinary Team Meetings Make a Difference in the Management of Lung Cancer? *Cancer* **2011**, *117*, 5112–5120. https://doi.org/10.1002/cncr.26149.
16. Freeman, R.K.; Ascioti, A.J.; Dake, M.; Mahidhara, R.S. The Effects of a Multidisciplinary Care Conference on the Quality and Cost of Care for Lung Cancer Patients. *Ann. Thorac. Surg.* **2015**, *100*, 1834–1838. https://doi.org/10.1016/j.athoracsur.2015.05.056.
18. Bilfinger, T.V.; Albano, D.; Perwaiz, M.; Keresztes, R.; Nemesure, B. Survival Outcomes Among Lung Cancer Patients Treated Using a Multidisciplinary Team Approach. *Clin. Lung Cancer* **2018**, *19*, 346–351. https://doi.org/10.1016/j.cllc.2018.01.006.
20. Pan, C.-C.; Kung, P.-T.; Wang, Y.-H.; Chang, Y.-C.; Wang, S.-T.; Tsai, W.-C. Effects of Multidisciplinary Team Care on the Survival of Patients with Different Stages of Non-Small Cell Lung Cancer: A National Cohort Study. *PLoS ONE* **2015**, *10*, e0126547. https://doi.org/10.1371/journal.pone.0126547.
24. Prado, C.M.; Purcell, S.A.; Laviano, A. Nutrition Interventions to Treat Low Muscle Mass in Cancer. *J. Cachexia Sarcopenia Muscle* **2020**, *11*, 366–380. https://doi.org/10.1002/jcsm.12525.
40. Brade, A.; Jao, K.; Yu, S.; Cheema, P.; Doucette, S.; Christofides, A.; Schellenberg, D. A Canadian Perspective on the Challenges for Delivery of Curative-Intent Therapy in Stage III Unresectable Non-Small Cell Lung Cancer. *Curr. Oncol.* **2021**, *28*, 1618–1629.
46. Muscaritoli, M.; Arends, J.; Bachmann, P.; Baracos, V.; Barthelemy, N.; Bertz, H.; Bozzetti, F.; Hütterer, E.; Isenring, E.; Kaasa, S.; et al. ESPEN Practical Guideline: Clinical Nutrition in Cancer. *Clin. Nutr.* **2021**, *40*, 2898–2913. https://doi.org/10.1016/j.clnu.2021.02.005.
50. Wang, J.; Tan, S.; Wu, G. Oral Nutritional Supplements, Physical Activity, and Sarcopenia in Cancer. *Curr. Opin. Clin. Nutr. Metab. Care* **2021**, *24*, 223–228. https://doi.org/10.1097/MCO.0000000000000736.
51. De Ruysscher, D.; Faivre-Finn, C.; Nackaerts, K.; Jordan, K.; Arends, J.; Douillard, J.Y.; Ricardi, U.; Peters, S. Recommendation for Supportive Care in Patients Receiving Concurrent Chemotherapy and Radiotherapy for Lung Cancer. *Ann. Oncol.* **2020**, *31*, 41–49. https://doi.org/10.1016/j.annonc.2019.10.003.
58. Blakely, K.; Cope, D.G. Establishing an Advanced Practice Nursing Clinic in the Cancer Setting. *Semin. Oncol. Nurs.* **2015**, *31*, 282–289. https://doi.org/10.1016/j.soncn.2015.08.004.
62. Friedman, E.L.; Kruklitis, R.J.; Patson, B.J.; Sopka, D.M.; Weiss, M.J. Effectiveness of a Thoracic Multidisciplinary Clinic in the Treatment of Stage III Non-Small-Cell Lung Cancer. *J. Multidiscip. Heal.* **2016**, *9*, 267–274.
63. Senter, J.; Hooker, C.; Lang, M.; Voong, K.R.; Hales, R.K. Thoracic Multidisciplinary Clinic Improves Survival in Patients with Lung Cancer. *Int. J. Radiat. Oncol. Biol. Phys.* **2016**, *96*, S134. https://doi.org/10.1016/j.ijrobp.2016.06.326.
72. Verma, V.; Simone, C.B.; Werner-Wasik, M. Acute and Late Toxicities of Concurrent Chemoradiotherapy for Locally-Advanced Non-Small Cell Lung Cancer. *Cancers* **2017**, *9*, E120. https://doi.org/10.3390/cancers9090120.
84. Patel, A.B.; Edelman, M.J.; Kwok, Y.; Krasna, M.J.; Suntharalingam, M. Predictors of Acute Esophagitis in Patients with Non–Small-Cell Lung Carcinoma Treated with Concurrent Chemotherapy and Hyperfractionated Radiotherapy Followed by Surgery. *Int. J. Radiat. Oncol. Biol. Phys.* **2004**, *60*, 1106–1112. https://doi.org/10.1016/j.ijrobp.2004.04.051.
85. Marks, L.B.; Yorke, E.D.; Jackson, A.; Ten Haken, R.K.; Constine, L.S.; Eisbruch, A.; Bentzen, S.M.; Nam, J.; Deasy, J.O. Use of Normal Tissue Complication Probability Models in the Clinic. *Int. J. Radiat. Oncol. Biol. Phys.* **2010**, *76* (Suppl. 3), S10–S19. https://doi.org/10.1016/j.ijrobp.2009.07.1754.
98. de van der Schueren, M.A.E. Use and Effects of Oral Nutritional Supplements in Patients with Cancer. *Nutrition* **2019**, *67–68*, 110550. https://doi.org/10.1016/j.nut.2019.07.002.
99. Cereda, E.; Cappello, S.; Colombo, S.; Klersy, C.; Imarisio, I.; Turri, A.; Caraccia, M.; Borioli, V.; Monaco, T.; Benazzo, M.; et al. Nutritional Counseling with or without Systematic Use of Oral Nutritional Supplements in Head and Neck Cancer Patients Undergoing Radiotherapy. *Radiother. Oncol.* **2018**, *126*, 81–88. https://doi.org/10.1016/j.radonc.2017.10.015.
100. Gomes, F.; Baumgartner, A.; Bounoure, L.; Bally, M.; Deutz, N.E.; Greenwald, J.L.; Stanga, Z.; Mueller, B.; Schuetz, P. Association of Nutritional Support With Clinical Outcomes Among Medical Inpatients Who Are Malnourished or at Nutritional Risk: An Updated Systematic Review and Meta-Analysis. *JAMA Netw. Open* **2019**, *2*, e1915138. https://doi.org/10.1001/jamanetworkopen.2019.15138.
101. Van Blarigan, E.L.; Fuchs, C.S.; Niedzwiecki, D.; Zhang, S.; Saltz, L.B.; Mayer, R.J.; Mowat, R.B.; Whittom, R.; Hantel, A.; Benson, A.; et al. Association of Survival With Adherence to the American Cancer Society Nutrition and Physical Activity Guidelines for Cancer Survivors After Colon Cancer Diagnosis: The CALGB 89803/Alliance Trial. *JAMA Oncol* **2018**, *4*, 783–790. https://doi.org/10.1001/jamaoncol.2018.0126.

102. Cereda, E.; Turri, A.; Klersy, C.; Cappello, S.; Ferrari, A.; Filippi, A.R.; Brugnatelli, S.; Caraccia, M.; Chiellino, S.; Borioli, V.; et al. Whey Protein Isolate Supplementation Improves Body Composition, Muscle Strength, and Treatment Tolerance in Malnourished Advanced Cancer Patients Undergoing Chemotherapy. *Cancer Med.* **2019**, *8*, 6923–6932. https://doi.org/10.1002/cam4.2517.
103. Fouladiun, M.; Körner, U.; Bosaeus, I.; Daneryd, P.; Hyltander, A.; Lundholm, K.G. Body Composition and Time Course Changes in Regional Distribution of Fat and Lean Tissue in Unselected Cancer Patients on Palliative Care—Correlations with Food Intake, Metabolism, Exercise Capacity, and Hormones. *Cancer* **2005**, *103*, 2189–2198. https://doi.org/10.1002/cncr.21013.
104. Pappalardo, G.; Almeida, A.; Ravasco, P. Eicosapentaenoic Acid in Cancer Improves Body Composition and Modulates Metabolism. *Nutrition* **2015**, *31*, 549–555. https://doi.org/10.1016/j.nut.2014.12.002.
105. Silva, M.; Bankier, A.A.; Centra, F.; Colombi, D.; Ampollini, L.; Carbognani, P.; Sverzellati, N. Longitudinal Evolution of Incidentally Detected Solitary Pure Ground-Glass Nodules on Ct: Relation to Clinical Metrics. *Diagn. Intervention. Radiol.* **2015**, *21*, 385–390. https://doi.org/10.5152/dir.2015.14457.
106. Murphy, R.A.; Mourtzakis, M.; Chu, Q.S.C.; Baracos, V.E.; Reiman, T.; Mazurak, V.C. Nutritional Intervention with Fish Oil Provides a Benefit over Standard of Care for Weight and Skeletal Muscle Mass in Patients with Nonsmall Cell Lung Cancer Receiving Chemotherapy. *Cancer* **2011**, *117*, 1775–1782. https://doi.org/10.1002/cncr.25709.
107. Sánchez-Lara, K.; Turcott, J.G.; Juárez-Hernández, E.; Nuñez-Valencia, C.; Villanueva, G.; Guevara, P.; De la Torre-Vallejo, M.; Mohar, A.; Arrieta, O. Effects of an Oral Nutritional Supplement Containing Eicosapentaenoic Acid on Nutritional and Clinical Outcomes in Patients with Advanced Non-Small Cell Lung Cancer: Randomised Trial. *Clin. Nutr.* **2014**, *33*, 1017–1023. https://doi.org/10.1016/j.clnu.2014.03.006.
108. Piraux, E.; Caty, G.; Aboubakar Nana, F.; Reychler, G. Effects of Exercise Therapy in Cancer Patients Undergoing Radiotherapy Treatment: A Narrative Review. *SAGE Open Med.* **2020**, *8*, 2050312120922657. https://doi.org/10.1177/2050312120922657.
109. Mustian, K.M.; Alfano, C.M.; Heckler, C.; Kleckner, A.S.; Kleckner, I.R.; Leach, C.R.; Mohr, D.; Palesh, O.G.; Peppone, L.J.; Piper, B.F.; et al. Comparison of Pharmaceutical, Psychological, and Exercise Treatments for Cancer-Related Fatigue A Meta-Analysis. *JAMA Oncol.* **2017**, *3*, 961–968. https://doi.org/10.1001/jamaoncol.2016.6914.
110. Peddle-McIntyre, C.J.; Singh, F.; Thomas, R.; Newton, R.U.; Galvão, D.A.; Cavalheri, V. Exercise Training for Advanced Lung Cancer. *Cochrane Database Syst. Rev.* **2019**, *2019*, CD012685. https://doi.org/10.1002/14651858.CD012685.pub2.
111. Salhi, B.; Haenebalcke, C.; Perez-Bogerd, S.; Nguyen, M.D.; Ninane, V.; Malfait, T.L.A.; Vermaelen, K.Y.; Surmont, V.F.; Van Maele, G.; Colman, R.; et al. Rehabilitation in Patients with Radically Treated Respiratory Cancer: A Randomised Controlled Trial Comparing Two Training Modalities. *Lung Cancer* **2015**, *89*, 167–174. https://doi.org/10.1016/j.lungcan.2015.05.013.
112. Lambert, J.E.; Hayes, L.D.; Keegan, T.J.; Subar, D.A.; Gaffney, C.J. The Impact of Prehabilitation on Patient Outcomes in Hepatobiliary, Colorectal, and Upper Gastrointestinal Cancer Surgery: A PRISMA-Accordant Meta-Analysis. *Ann. Surg.* **2021**, *274*, 70–77. https://doi.org/10.1097/SLA.0000000000004527.
113. Morton, R.W.; Murphy, K.T.; McKellar, S.R.; Schoenfeld, B.J.; Henselmans, M.; Helms, E.; Aragon, A.A.; Devries, M.C.; Banfield, L.; Krieger, J.W.; et al. A Systematic Review, Meta-Analysis and Meta-Regression of the Effect of Protein Supplementation on Resistance Training-Induced Gains in Muscle Mass and Strength in Healthy Adults. *Br. J. Sports Med.* **2018**, *52*, 376–384. https://doi.org/10.1136/bjsports-2017-097608.
114. Markofski, M.M.; Jennings, K.; Timmerman, K.L.; Dickinson, J.M.; Fry, C.S.; Borack, M.S.; Reidy, P.T.; Deer, R.R.; Randolph, A.; Rasmussen, B.B.; et al. Effect of Aerobic Exercise Training and Essential Amino Acid Supplementation for 24 Weeks on Physical Function, Body Composition, and Muscle Metabolism in Healthy, Independent Older Adults: A Randomized Clinical Trial. *J. Gerontol. A Biol. Sci. Med. Sci.* **2019**, *74*, 1598–1604. https://doi.org/10.1093/gerona/gly109.
115. National Comprehensive Cancer Network (NCCN). NCCN Guidelines—Non-Small Cell Lung Cancer Version 4.2023. Available online: https://www.nccn.org/guidelines/recently-published-guidelines (accessed on 8 October 2023).
116. Borkenhagen, J.F.; Bergom, C.; Rapp, C.T.; Klawikowski, S.J.; Rein, L.E.; Gore, E.M. Dosimetric Predictors of Cardiotoxicity in Thoracic Radiotherapy for Lung Cancer. *Clin. Lung Cancer* **2019**, *20*, 435–441. https://doi.org/10.1016/j.cllc.2019.05.014.
117. Yazbeck, V.Y.; Villaruz, L.; Haley, M.; Socinski, M.A. Management of Normal Tissue Toxicity Associated with Chemoradiation (Primary Skin, Esophagus, and Lung). *Cancer J.* **2013**, *19*, 231–237. https://doi.org/10.1097/PPO.0b013e31829453fb.

118. Murray, P. The Pathway Study: Results of a Pilot Feasibility Study in Patients Suspected of Having Lung Carcinoma Investigated in a Conventional Chest Clinic Setting Compared to a Centralised Two-Stop Pathway. *Lung Cancer* **2003**, *42*, 283–290. https://doi.org/10.1016/S0169-5002(03)00358-1.
119. Riedel, R.F.; Wang, X.; McCormack, M.; Toloza, E.; Montana, G.S.; Schreiber, G.; Kelley, M.J. Impact of a Multidisciplinary Thoracic Oncology Clinic on the Timeliness of Care. *J. Thorac. Oncol.* **2006**, *1*, 692–696.
120. Bydder, S.; Nowak, A.; Marion, K.; Phillips, M.; Atun, R. The Impact of Case Discussion at a Multidisciplinary Team Meeting on the Treatment and Survival of Patients with Inoperable Non-Small Cell Lung Cancer. *Intern. Med. J.* **2009**, *39*, 838–841. https://doi.org/10.1111/j.1445-5994.2009.02019.x.
121. Freeman, R.K.; Van Woerkom, J.M.; Vyverberg, A.; Ascioti, A.J. The Effect of a Multidisciplinary Thoracic Malignancy Conference on the Treatment of Patients with Lung Cancer☆. *Eur. J. Cardiothorac. Surg.* **2010**, *38*, 1–5. https://doi.org/10.1016/j.ejcts.2010.01.051.
122. Rogers, M.J.; Matheson, L.; Garrard, B.; Maher, B.; Cowdery, S.; Luo, W.; Reed, M.; Riches, S.; Pitson, G.; Ashley, D.M. Comparison of Outcomes for Cancer Patients Discussed and Not Discussed at a Multidisciplinary Meeting. *Public. Health* **2017**, *149*, 74–80. https://doi.org/10.1016/j.puhe.2017.04.022.
123. Stone, E.; Rankin, N.; Kerr, S.; Fong, K.; Currow, D.C.; Phillips, J.; Connon, T.; Zhang, L.; Shaw, T. Does Presentation at Multidisciplinary Team Meetings Improve Lung Cancer Survival? Findings from a Consecutive Cohort Study. *Lung Cancer* **2018**, *124*, 199–204. https://doi.org/10.1016/j.lungcan.2018.07.032.
124. Friedman, E.L.; Szwerc, M.F.; Kruklitis, R.; Weiss, M.J. Value of Thoracic Multidisciplinary Clinic (TMDC) in Determining Appropriate Treatment of Stage III Non-Small Cell Lung Cancer (NSCLC). *J. Clin. Oncol.* **2013**, *31*, 82.
125. Harbegue, K.; Mejri, N.; El Benna, H.; Labidi, S.; Daoud, N.; Boussen, H. Challenges in the Management of Stage III Non-Small Cell Lung Cancer (NSCLC) within Multidisciplinary Team (MDT): A Lung Cancer Center Experience. *Ann. Oncol.* **2019**, *30*, ii35.
126. Hung, H.-Y.; Tseng, Y.-H.; Chao, H.-S.; Chiu, C.-H.; Hsu, W.-H.; Hsu, H.-S.; Wu, Y.-C.; Chou, T.-Y.; Chen, C.-K.; Lan, K.-L.; et al. Multidisciplinary Team Discussion Results in Survival Benefit for Patients with Stage III Non-Small-Cell Lung Cancer. *PLoS ONE* **2020**, *15*, e0236503. https://doi.org/10.1371/journal.pone.0236503.
127. Hergebue, K.; Mejri, N.; Berrazega, Y.; Rachdi, H.; El Benna, H.; Labidi, S.; Boussen, H. Therapeutic Results and Prognostic Factors of Stage III NSCLC: A Population-Based Study in Tunisia. *Exp. Oncol.* **2021**, *43*, 61–66. https://doi.org/10.32471/exp-oncology.2312-8852.vol-43-no-1.15787.
